# Heterologous xylose isomerase pathway and evolutionary engineering improve xylose utilization in *Saccharomyces cerevisiae*

**DOI:** 10.3389/fmicb.2015.01165

**Published:** 2015-10-21

**Authors:** Xin Qi, Jian Zha, Gao-Gang Liu, Weiwen Zhang, Bing-Zhi Li, Ying-Jin Yuan

**Affiliations:** ^1^Key Laboratory of Systems Bioengineering, Ministry of Education, Tianjin UniversityTianjin, China; ^2^SynBio Research Platform, Collaborative Innovation Center of Chemical Science and Engineering (Tianjin), School of Chemical Engineering and Technology, Tianjin UniversityTianjin, China

**Keywords:** synthetic biology, xylose isomerase, XylA, xylose utilization, yeast, evolutionary engineering

## Abstract

Xylose utilization is one key issue for the bioconversion of lignocelluloses. It is a promising approach to engineering heterologous pathway for xylose utilization in *Saccharomyces cerevisiae*. Here, we constructed a xylose-fermenting yeast SyBE001 through combinatorial fine-tuning the expression of *XylA* and endogenous *XKS1*. Additional overexpression of genes *RKI1, RPE1, TKL1*, and *TAL1* in the non-oxidative pentose phosphate pathway (PPP) in SyBE001 increased the xylose consumption rate by 1.19-fold. By repetitive adaptation, the xylose utilization rate was further increased by ∼10-fold in the resultant strain SyBE003. Gene expression analysis identified a variety of genes with significantly changed expression in the PPP, glycolysis and the tricarboxylic acid cycle in SyBE003.

## Introduction

Efficient utilization of xylose is critical for economical cellulosic ethanol production because xylose is the second abundant sugar in lignocellulosic hydrolysates (Li et al., 2010; Qin et al., 2012). Xylose metabolic pathways from natural xylose-fermenting microorganisms have been heterologously expressed in *Saccharomyces cerevisiae*, which is one of the most promising microbes for cellulosic ethanol production (Zha et al., 2012, 2014). However, the utilization of xylose is much slower than glucose utilization in engineered yeasts (Wang et al., 2014; Shen et al., 2015). Improvement of xylose utilization in engineered yeasts is an urge need for efficient conversion of cellulosic resources.

The xylose reductase/xylitol dehydrogenase (XR/XDH) pathway from fungus, one of the two available ways so far, converts xylose into xylulose catalyzed by NADPH-dependent xylose reductase and NAD^+^-preferred xylitol dehydrogenase with xylitol as the by-product. The xylose isomerase (XI) pathway converts xylose to xylulose by XI encoded by the gene *XylA*. In both pathways, xylulose is then channeled into the glycolytic pathway through the non-oxidative pentose phosphate pathway (PPP) to produce ethanol under anaerobic conditions. Several approaches have been applied to optimize the XR/XDH pathway to reduce xylitol production and increase ethanol yield, including protein engineering of the cofactor preference of XR or XDH for cofactor recycling, overexpression of the genes in the PPP, and heterologous expression of xylose-specific transporters (Jeppsson et al., 2006; Karhumaa et al., 2007a; Matsushika et al., 2008; Petschacher and Nidetzky, 2008; Bengtsson et al., 2009; Runquist et al., 2009). As an alternative, the XI pathway bypasses the cofactor imbalance and can achieve a much higher ethanol yield close to the theoretical value (Kuyper et al., 2003; Brat et al., 2009; Ha et al., 2011; Lee et al., 2014). However, the overall xylose utilization rate by the XI pathway in yeast is notably lower than that by the XR/XDH pathway, and so efforts are required to increase the xylose utilization rate by this pathway (Kuyper et al., 2003; Chu and Lee, 2007; Karhumaa et al., 2007b; Brat and Boles, 2013; Lee et al., 2014).

Rational metabolic modifications to accelerate xylose uptake and metabolism were employed in recombinant *S. cerevisiae* expressing the XI pathway (Kuyper et al., 2005a; Lee et al., 2012; Zhou et al., 2012; Nijland et al., 2014; Wang et al., 2015). (*i*) One or all genes encoding the enzymes in the non-oxidative PPP were over-expressed in *S. cerevisiae* to increase the flux toward the glycolytic pathway; (*ii*) *XKS1* encoding xylulose kinase responsible for phosphorylation of xylulose was over-expressed to improve xylose fermentation; (*iii*) Directed evolution of a XI was conducted to accelerate xylose utilization; and (*iv*) Heterologous xylose-specific transporters were over-expressed to enhance xylose transport. Efficient xylose utilization was achieved in the strain RWB217 by overexpression of *XylA, XKS1* and the four genes of *RPE1, RKI1, TAL1*, and *TKL1* in the PPP together with deletion of *GRE3* (Kuyper et al., 2005a). However, these modifications did not visibly improve xylose utilization in other host yeasts for unknown reasons (Karhumaa et al., 2007b; Zhou et al., 2012). Thus, alternative approaches need to be introduced to improve xylose utilization in recombinant *S. cerevisiae.*

Evolutionary engineering was an efficient approach to improving the utilization of non-favored carbon sources such as arabinose and galactose in *S. cerevisiae* (Wisselink et al., 2010; Hong et al., 2011; Scalcinati et al., 2012; Demeke et al., 2015). Similarly, such a strategy has been successfully applied to evolve the XI pathway for accelerated xylose fermentation (Kuyper et al., 2005b; Zhou et al., 2012; Lee et al., 2014).

In this study, a combinatorial design was used to modulate the expression of *XylA* from fungus *Piromyces* and endogenous *XKS1* in *S. cerevisiae*. The rational engineering for the non-oxidative PPP and evolutionary engineering resulted in an efficient xylose-utilizing strain.

## Materials and Methods

### Strains and Media

*Escherichia coli* DH5*α* was used for plasmid construction in this study and was grown in Luria-Bertani medium with 100 mg/L ampicillin. Yeast strains used in the study are listed in **Table 1**. YNB medium with 20 g/L glucose was used in the construction of recombinant yeasts (Zha et al., 2013). Agar plates were prepared by the addition of 20 g/L agar. Adaptive evolution was performed in YNB medium with 20 g/L xylose. The fermentation was conducted in YPX medium (10 g/L yeast extract, 20 g/L peptone, and 20 g/L or 40 g/L xylose) or YPGX medium (10 g/L yeast extract, 20 g/L peptone, 20 g/L glucose, and 20 g/L xylose).

**Table 1:** The strains and plasmids used in this study.

Strain	Genotype	Sources
L2612	MATalpha,*leu2*,*ura3, trp1*	Jin et al., 2003
SyBE001	L2612,*ura3:*:[TDH1p-XKS1,pRS425-TDH3p- XylA]	This study
SyBE002	SyBE001, *AUR1*:: [TDH1p-RPE1,PGK1p-TAL1], *trp1*:: [PGK1p-RKI1,TDH3p-TKL1]	This study
SyBE003	Evolved from SyBE002	This study
tkl2Δ	Yeast knock-out strain	Open biosystems
SyBE002-TKL2Δ	SyBE002, *tkl2::*KanMX	This study
**Plasmid**		
pTDH1XK	YIplac211, *TDH1p-XKS1*	This study
pTDH3XK	YIplac211, *TDH3p-XKS1*	This study
pPGK1XK	YIplac211, *PGK1p-XKS1*	This study
pTDH3XI	pRS425, *TDH3p-XylA* (*Piromyces*)	This study
pHXK2XI	pRS425, *HXK2p-XylA* (*Piromyces*)	This study
pPGK1XI	pRS425, *PGK1p-XylA* (*Piromyces*)	This study
pAUR101-RPE1-TAL1	pAUR101, *TDH1p-RPE1-PGK1t, PGK1p-TAL1-PGK1t*	This study
pRS304-RKI1-TKL1	pRS304, *PGK1p-RKI1-PGK1t, TDH3p-TKL1-PGK1t*	This study

### Plasmid Construction and Transformation

#### Plasmid Construction

Gene *XylA* from *Piromyces* sp. *E2* (ATCC 76762) was optimized according to codon preference of *S. cerevisiae*. The synthesized *XylA* (Supplementary Table S1) was inserted into plasmid pRS425, followed by the insertion of the promoters *TDH3p, HXK2p*, or *PGK1p* to form the plasmids pTDH3XI, pHXK2XI, and pPGK1XI, respectively.

To construct the *XKS1* cassette, the *XKS1* and *PGK1* terminator were amplified from the genomic DNA of strain L2612, respectively, and assembled by fusion PCR. The fused fragment was then inserted into plasmid YIplac211. Promoters *TDH1p, TDH3p*, or *PGK1p* were inserted in front of *XKS1* gene to form three *XKS1* cassettes, resulting in plasmids pTDH1XK, pTDH3XK, and pPGK1XK, respectively.

The modules for overexpression of *RPE1, RKI1, TAL1*, and *TKL1* were constructed as follows. The individual expression cassettes, namely *TDH1p-RPE1-PGK1t, PGK1p-TAL1-PGK1t, PGK1p-RKI1-PGK1t*, and *TDH3p-TKL1-PGK1t*, were obtained by fusion PCR of genomic DNA of yeast L2612. The expression cassettes *TDH1p-RPE1-PGK1t* and *PGK1p-TAL1-PGK1t* were then combined together using fusion PCR and inserted into plasmid pAUR101 (Takara Bio, Kyoto, Japan), creating plasmid pAUR101-RPE1-TAL1. Cassettes *PGK1p-RKI1-PGK1t* and *TDH3p-TKL1-PGK1t* were assembled and cloned into plasmid pRS304, generating plasmid pRS304-RKI1-TKL1.

To knockout gene *TKL2* in SyBE002, the *TKL2* knockout box was amplified from the genomic DNA of the strain tkl2Δ from the strain collection of single-gene deletion (Open Biosystems, Huntsville, Ala, USA). The constructed knockout DNA fragment was used directly for transformation into SyBE002.

All the primers used in this study were listed in Supplementary Table S2.

#### Transformation

The transformants of plasmids pTDH3XI, pHXK2XI, pPGK1XI, pTDH1XK, pTDH3XK, pPGK1XK, and pRS304-RKI1-TKL1 were selected in YNB medium with 20 g/L glucose lacking specific amino acids. Integration of pAUR101-RPE1-TAL1 was performed in YNB plate supplemented with 0.5 mg/L Aureobasidin A (Takara Bio, Kyoto, Japan). The *TKL2*-knockout strain was selected on YNB plate with 200 mg/L G418 (Merck, Darmstadt, Germany). All the transformation was conducted according to the standard procedures (Gietz et al., 1995).

### Adaptive Evolution on Xylose

The evolution was carried out in 50 mL YNB medium with 20 g/L xylose in 250 mL flasks at 30°C and 150 rpm under oxygen-limited conditions. For each adaptation cycle, growth was initiated with the cell density of 0.2 (OD_600_). Further evolution cycles were repeated with the identical conditions and ceased until the constant cell density. After the evolution of about 40 transfers in 75 days, cells from the shaking flasks were streaked on YNB plates with 20 g/L xylose for isolation of a single colony. Twenty colonies with the biggest sizes were chosen for additional analysis. The selected colonies were first cultivated in YNB medium containing 20 g/L glucose for 2 days, and then 100-μL culture was taken and inoculated into 3-mL YNB medium containing 20 g/L xylose in rubber-sealed 15-ml culture tubes and cultivated at 30°C and 150 rpm. The cell density and metabolites were analyzed after 72 h.

### Anaerobic Fermentation

Yeast seeds were cultivated in YNB medium with 20 g/L glucose at 30°C and 200 rpm and inoculated into fresh media when cells reached mid-log phase. Anaerobic fermentation was performed in 100 mL of YPX or YPGX in 250-mL flasks sealed by a rubber stopper with a needle to release CO_2_ produced during fermentation. Flasks were incubated in a rotary shaker (Honor, Tianjin, China) with shaking (150 rpm) at 30°C. Initial cell densities were 1.0 of OD_600_ for the fermentation with 20 g/L xylose, and 2.0 of OD_600_ was used for other fermentation. Cell density was monitored by measuring the OD_600_ using a 756 spectrophotometer (Kanasi, Tianjin, China). The experiments were repeated twice independently.

### Analysis of Sugars and Fermentation Products

Sugars and fermentation products were analyzed using an HPLC system consisting of a Waters 1515 pump (Milford, MA, USA) and a Waters 2414 refractive index detector. The substances were separated on an Aminex HPX-87H carbohydrate analysis column (Bio-Rad, Hercules, CA, USA) at 65°C using 5 mM sulphuric acid as the mobile phase with a flow rate of 0.6 mL/min.

### Transcriptional Analysis by Quantitative RT-PCR

After anaerobic growth on 20 g/L xylose for 18 h, the cells were harvested by centrifugation (4,000 rpm) for 5 min at 4°C, followed by two washes with ice-cold water. Then cells were stored in liquid nitrogen before use. Total RNA was extracted by Mini RNA dropout kit (Tiangen, Beijing, China). RNA integrity and quality were verified by RNA electrophoresis and NanoDrop-1000 (Thermo Scientific, Wilmington, DE, USA). Totally 1 μg of RNA was used for each reverse transcription with the Reverse Transcription Kit (Tiangen, Beijing, China). Transcription was performed for 1 h at 37°C. The quantitative measurement was carried out using RealMaster Mix Kit (Tiangen, Beijing, China). For each reaction, a total of 20 μL was used consisting of 9 μL RealMaster Mix buffer, 0.5 μL each of forward and reverse primer (10 μM each), 0.5 μL cDNA template, and 9.5 μL ddH_2_O. The primers (Supplementary Table S3) were designed according to the sequences from Saccharomyces Genome Database (http://www.yeastgenome.org). Quantitative RT-PCR was run in an ABI7300 Thermo cycler and the conditions employed were as follows: 95°C for 2 min; 94°C for 15 s, 60°C for 30 s, and 72°C for 40 s (40 cycles). Three biological replicates were performed for each gene. The threshold cycle value (Ct) for each sample was determined using the ABI7300 software. The data was normalized using the gene *ACT1* as the internal standard and analyzed according to the 2*^-ΔΔCT^* method (Schmittgen and Livak, 2008). ΔCT was obtained by subtracting the Ct values of *ACT1* from the Ct values of the gene of interest. ΔΔCT was then calculated by subtracting mean ΔCT of the samples in SyBE002 from mean ΔCT of samples in SyBE003. Fold changes of gene expression were calculated by the equation of 2*^-^*^ΔΔ^*^CT^*.

### Statistical Analysis

Student’s *t*-test was introduced to statistically analyze the data using statistical function tools of Microsoft Excel 2010. Fisher’s exact test and Pearson’s χ^2^ test were applied to determine the significant association between the expression levels in SyBE002 and those in SyBE003. All tests of statistical significance were two sided. *P* < 0.05 were considered statistically significant.

## Results

### Genetic Construction of Xylose-fermenting Yeasts Expressing the Xylose Isomerase Pathway

**FIGURE 1:**
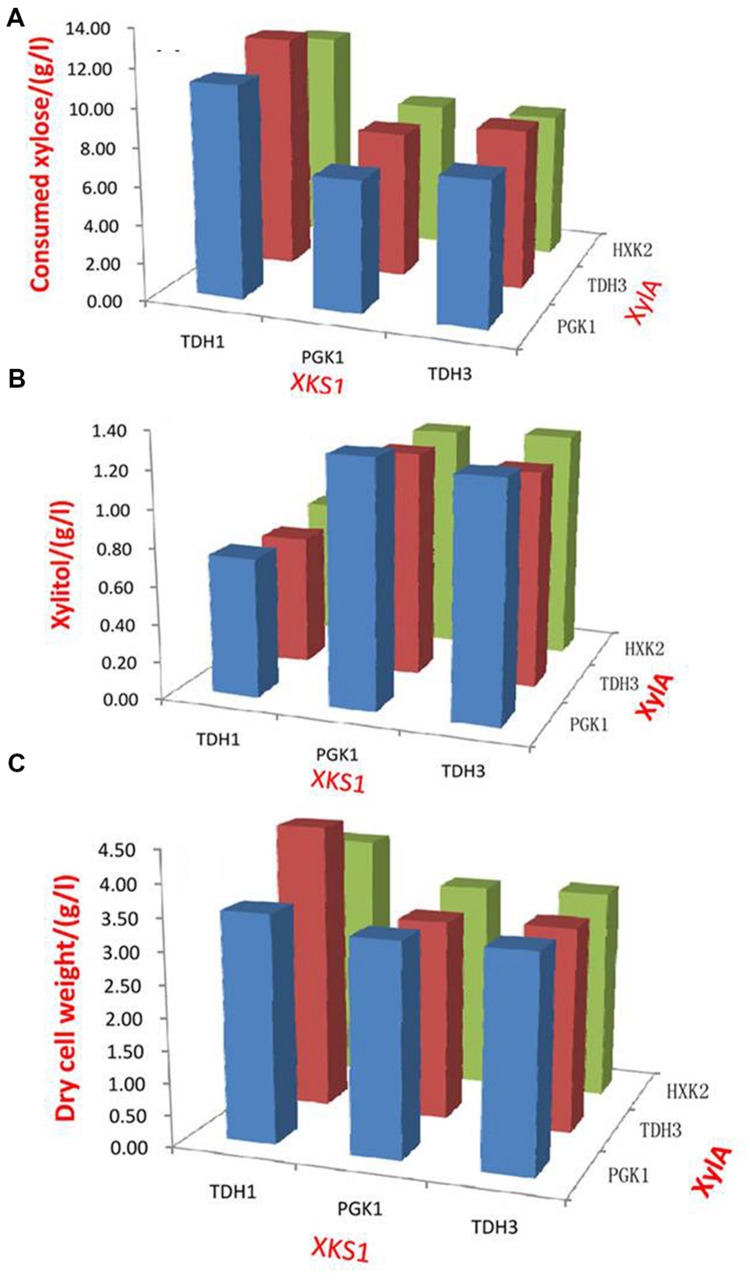
**Xylose consumption (A), xylitol production (B), and cell growth (C) of strains expressing different modules of XylA-XKS1.** Promoters TDH1, PGK1, and TDH3 were applied to control the expression of XKS1. Promoters PGK1, TDH3, and HXK2 were used to express XylA. Set the expression strength of promoter HXK2p as 1 unit, the expression strengths of TDH1p, TDH3p and PGK1p are calculated to be ∼2.5, ∼9, and ∼6 units based on a previous study, respectively (Lu and Jeffries, 2007). In total, 9 (3 × 3) strains were constructed and evaluated for the oxygen-limited fermentation on xylose, conducted in 50 mL YPX in 250 mL flasks with the initial cell density at 0.5 of OD600. The samples were harvested at 72 h and analyzed of the OD_600_ values and components. The dry cell weight (DCW) was calculated from OD_600_ with the coefficient of 0.526 g DCW/L ⋅ OD_600_.

The enzyme products of *XylA* and *XKS1* catalyze the formation of 5-phosphate-xylulose that was metabolized by the following non-oxidative PPP (Jin et al., 2003; Lonn et al., 2003). Balancing the expression of *XylA* and *XKS1* can be a way to optimize xylose metabolic pathway and improve xylose utilization. We evaluated the effects of different promoters on balancing the expression of *XylA* and *XKS1*. The promoters with different transcriptional strengths were applied to tune the expression of *XylA* and *XKS1.* Promoters *TDH3p, HXK2p*, and *PGK1p* were applied to express *XylA* in a multicopy plasmid pRS425 (Lu and Jeffries, 2007). Promoters *TDH1p, TDH3p*, and *PGK1p* were used to control the expression of *XKS1*. This strategy generated nine recombinant strains with different expression levels of *XylA* and *XKS1* (**Figure 1**).

These strains were then comparatively characterized in terms of cell growth, xylose consumption, and xylitol production under oxygen-limited conditions (**Figure 1**, Supplementary Table S4). The strains with *XKS1* under the control of *TDH1p* showed higher abilities of xylose utilization than those with *XKS1* controlled by *TDH3p* or *PGK1p* (**Figure 1A**). Xylitol production in this group was also much lower than other two groups (**Figure 1B**). In addition, the cell densities of the cultures were highest in the strains with *XKS1* controlled by *TDH1p* (**Figure 1C**).

Expression of *XylA* had little effect on xylose utilization. When *XKS1* was expressed by *TDH1* promoter, the strain with *XylA* controlled by *TDH3p*, named SyBE001, showed a slightly higher xylose utilization rate and cell density (**Figures 1A,C**). The xylose consumption rate of SyBE001 was 8.9%-79.4% higher than other eight strains (**Figure 1A**). Thus, we genetically constructed an optimal recombinant *S. cerevisiae* through fine-tuning the expression of *XylA* and *XKS1*.

**FIGURE 2:**
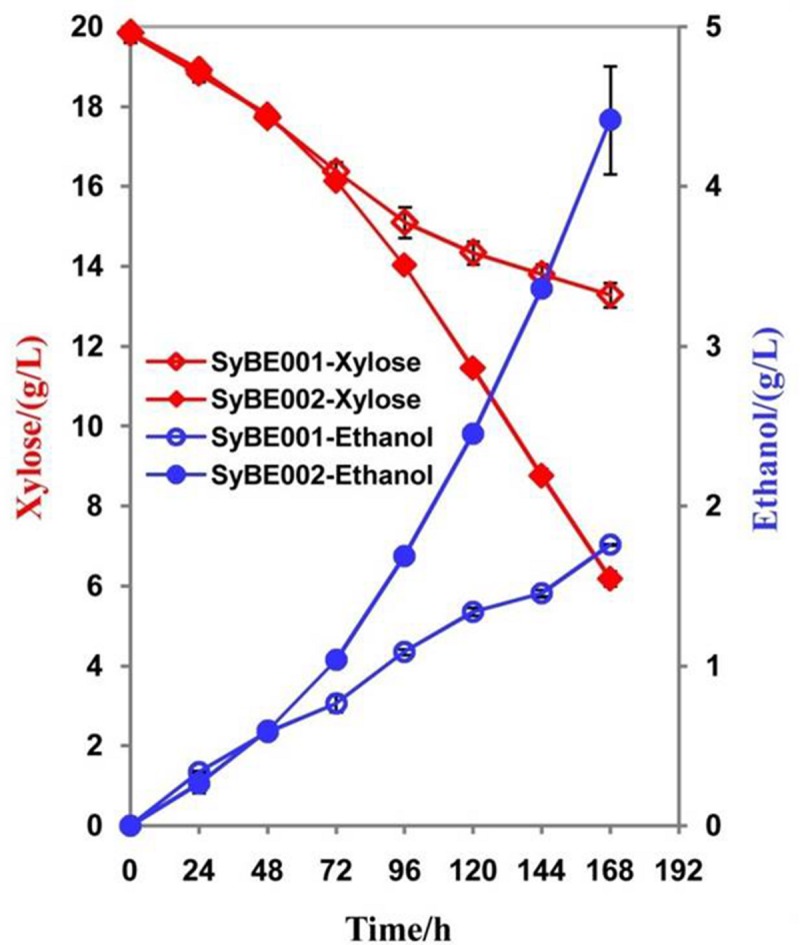
**Comparison of xylose fermentation by strain SyBE001 and strain SyBE002.** In strain SyBE002, genes RPE1, RKI1, TKL1, and TAL1 in the non-oxidative PPP were over-expressed while they were not engineered in strain SyBE001. The xylose fermentations were conducted in 100 mL YPX in 250 mL flasks with the beginning cell density adjusted to 1.0 of OD_600_. The calculation of xylose consumption rate was conducted at 168 h.

In the strain SyBE001, the xylose consumption rate was 0.037 g/L/h. The ethanol yield and xylitol yield was 0.28 and 0.19 g/g, respectively. This might be ascribed to the limited flux through the non-oxidative PPP. Enzymes of the non-oxidative PPP are considered to be rate-limiting in xylose metabolism (Karhumaa et al., 2005; Kuyper et al., 2005a; Chu and Lee, 2007). To eliminate these bottlenecks, we over-expressed genes *RPE1, RKI1, TAL1*, and *TKL1* in SyBE001, resulting in strain SyBE002. As shown in Supplementary Figure S1, the expression of the genes was enhanced by 40–134-fold in SyBE002. The fermentation performances of SyBE001 and SyBE002 were also compared to examine the roles of *RPE1, RKI1, TAL1*, and *TKL1* (**Figure 2**). Compared with strain SyBE001, the xylose consumption rate of SyBE002 increased by 1.19-fold and the ethanol production was enhanced by 1.51-fold accordingly. Meanwhile, xylitol yield and glycerol yield decreased by 21% and 43%, respectively. The results demonstrated that overexpression of genes in the non-oxidative PPP could significantly improve xylose fermentation. However, the resultant strain SyBE002 consumed xylose still at a low rate, and therefore further modification is needed to achieve rapid ethanol production from xylose.

### Evolutionary Engineering of SyBE002 on Xylose

To accelerate xylose metabolism in SyBE002, evolutionary engineering was applied. After a 75-day evolution conducted under oxygen-limited conditions, the candidate colonies were selected based on colony sizes as reported before (Du et al., 2012; Kim et al., 2013). Twenty biggest colonies were picked and evaluated in terms of xylose consumption, and the colony exhibiting the highest xylose consumption rate was designated as SyBE003 and investigated further.

### Fermentation of SyBE002 and SyBE003 on Xylose

**FIGURE 3:**
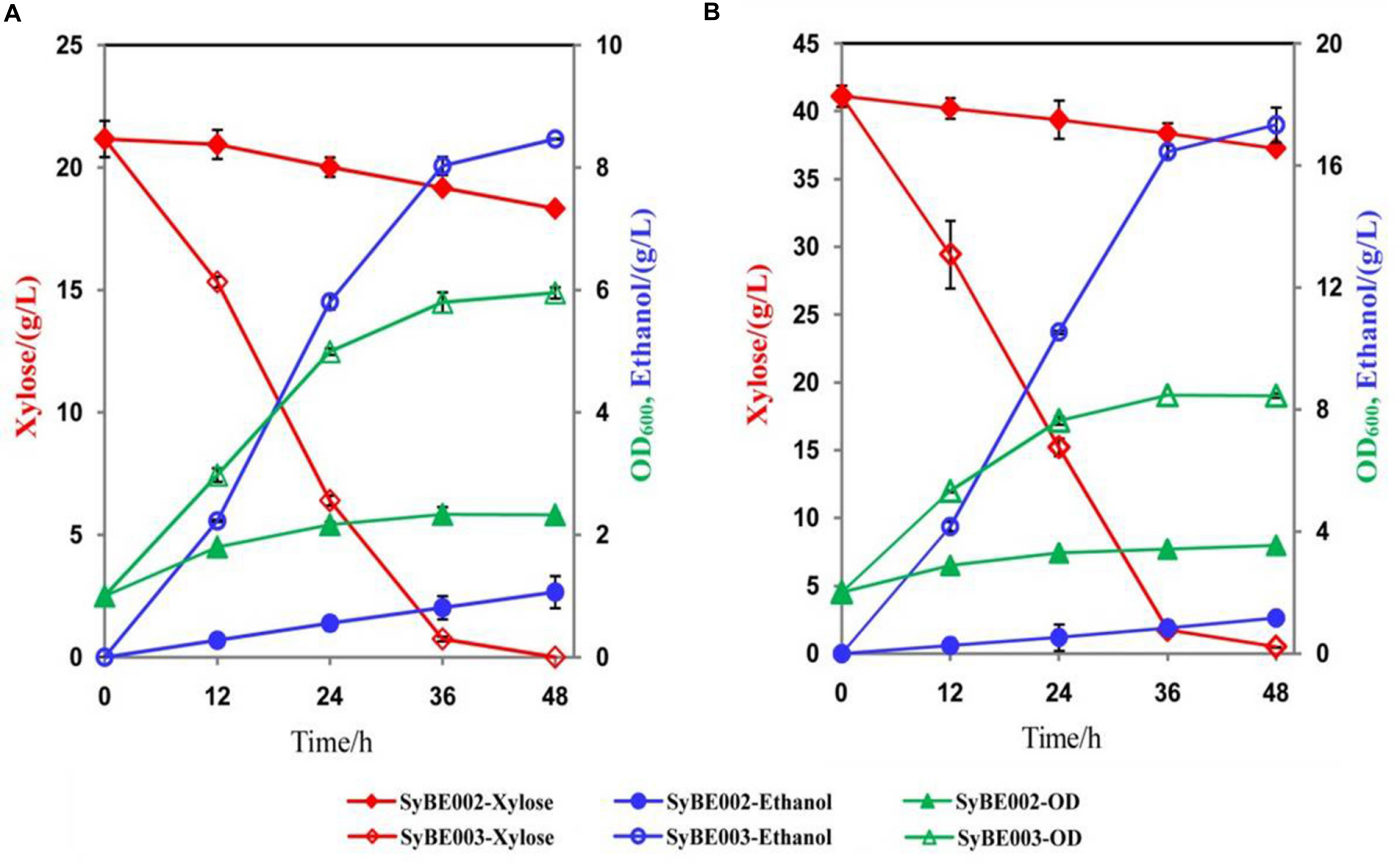
**The ethanol fermentation by SyBE002 and SyBE003 on 20 g/L xylose (A) and 40 g/L xylose (B).** The initial cell density was adjusted to 1.0 of OD600. The fermentations were performed in 100 mL medium in 250-mL Erlenmeyer flasks. The data was the mean ± SD of the duplicated experiments. The calculation of xylose consumption rate was performed at 48 h.

To compare their abilities to produce ethanol from xylose, the anaerobic fermentation of SyBE002 and SyBE003 was first carried out on 20 g/L xylose (**Figure 3**). During the first 36 h, strain SyBE003 metabolized more than 95% of the total xylose, while strain SyBE002 consumed only 7% (**Figure 3A**). After 48 h, SyBE003 finished consumption of all xylose and produced 8.47 g/L ethanol with a yield of 0.40 g/g xylose, while SyBE002 consumed only 10% of the total xylose (**Table 2**; **Figure 3A**), indicating that xylose utilization was increased more than 10 times after adaptation. The xylitol yield in SyBE003 was 0.01 g/g, indicating that almost all the assimilated xylose was utilized instead of being secreted as a byproduct, while strain SyBE002 had a 14-fold higher xylitol yield (**Table 2**). In addition, the glycerol yield in SyBE003 was 0.05 g/g xylose, which was 37.5% lower than that of SyBE002 (**Table 2**).

**Table 2:** Summary of the anaerobic batch fermentation by strain SyBE002 and the evolved strain SyBE003 on different carbon sources.

	SyBE002	SyBE003	SyBE002	SyBE003	SyBE002	SyBE003
Carbon source	2% xylose	2% xylose	4% xylose	4% xylose	2% xylose+2% glucose	2% xylose+2% glucose
Ethanol yield (g/g)	0.30 ± 0.03^a^	0.40 ± 0.00^a^	0.29 ± 0.00^a^	0.43 ± 0.01^a^	0.38 ± 0.01^b^	0.41 ± 0.02^b^
Xylitol yield (g/g)	0.15 ± 0.00^a^	0.01 ± 0.00^a^	0.18 ± 0.00^a^	0.02 ± 0.00^a^	0.14 ± 0.02^b^	0.01 ± 0.00^b^
Glycerol yield (g/g)	0.08 ± 0.00^a^	0.05 ± 0.00^a^	0.09 ± 0.01^a^	0.05 ± 0.01^a^	0.05 ± 0.00^b^	0.04 ± 0.00^b^

*Values are presented as the average and standard deviation of two independent experiments.*

*^a^The values were calculated by the concentrations of the metabolites measured at 48 h.*

*^b^The values were analyzed based on total sugars determined at 36 h in the fermentation.*

The performance of SyBE003 was also examined under a higher concentration of xylose (40 g/L) (**Figure 3**). To achieve a higher ethanol productivity, the initial cell density was elevated to 2.0 of OD_600_ (**Figure 3B**). After 36 h, 96% xylose was consumed by SyBE003, and the xylose consumption rate increased up to ∼1 g/L/h. In contrast, only 5% xylose was utilized by SyBE002. The xylitol yield and glycerol yield in SyBE003 decreased by eightfold and 44% compared to those in SyBE002, respectively. Consistently, the ethanol yield in SyBE003 (0.43 g/g) was 48% higher than that in SyBE002 (0.29 g/g) (**Table 2**). These results clearly demonstrated that the performance of the evolved strain SyBE003 on xylose was significantly improved after evolution.

### Fermentation of SyBE002 and SyBE003 on Mixed Sugars

**FIGURE 4:**
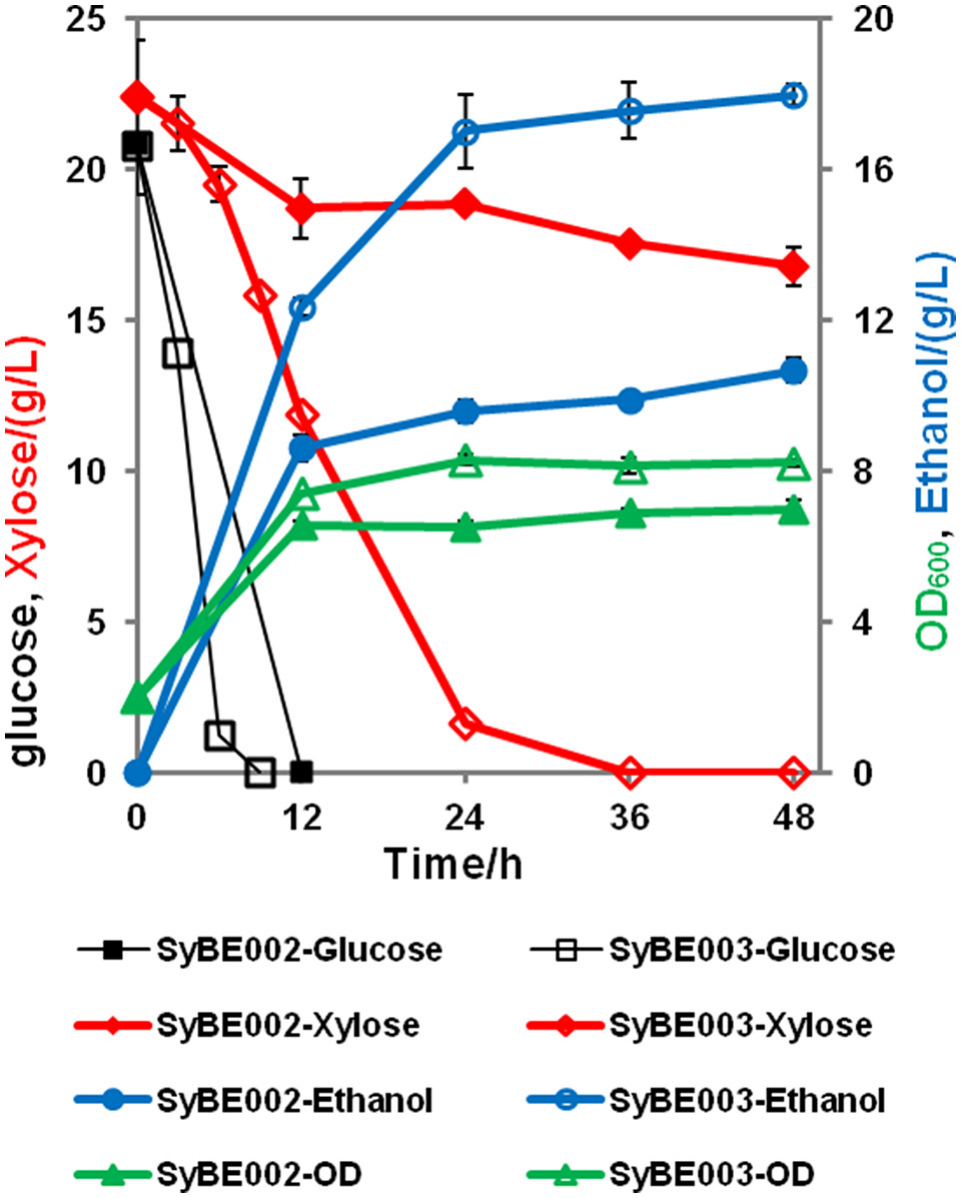
**Anaerobic batch fermentation by SyBE002 and SyBE003 on mixed xylose and glucose, 20 g/L each.** The initial cell density of inocula was 2.0 of OD_600_. The fermentations were carried out in 100 mL medium in 250-mL Erlenmeyer flasks. The data was the mean ± SD of the duplicated experiments. The calculation of xylose consumption rate was carried out at 48 h.

To achieve economical feasibility of ethanol fermentation on lignocellulosic hydrolysates, efficient co-utilization of xylose, and glucose is necessary. Thus, we characterized the performance of SyBE003 on mixed sugars. As shown in **Figure 4**, glucose was preferably consumed by both SyBE002 and SyBE003 during the first 12 h followed by the utilization of xylose. In SyBE003, 93% xylose was consumed in 24 h, while only 16% of the xylose was utilized by SyBE002. The final ethanol yield in SyBE003 reached 0.41 g/g sugar, 8% higher than that in SyBE002 (0.38 g/g sugar) (**Figure 4**). The xylitol yield of SyBE003 was 13-fold lower than that of SyBE002 whereas the glycerol yield of both strains was almost the same (**Table 2**). Taken together, the results demonstrated that the xylose-fermenting *S. cerevisiae* SyBE003 obtained through evolutionary engineering was an efficient ethanol producer on mixed sugars of glucose and xylose.

### The Expression Profile of Genes in Xylose Metabolism

**FIGURE 5:**
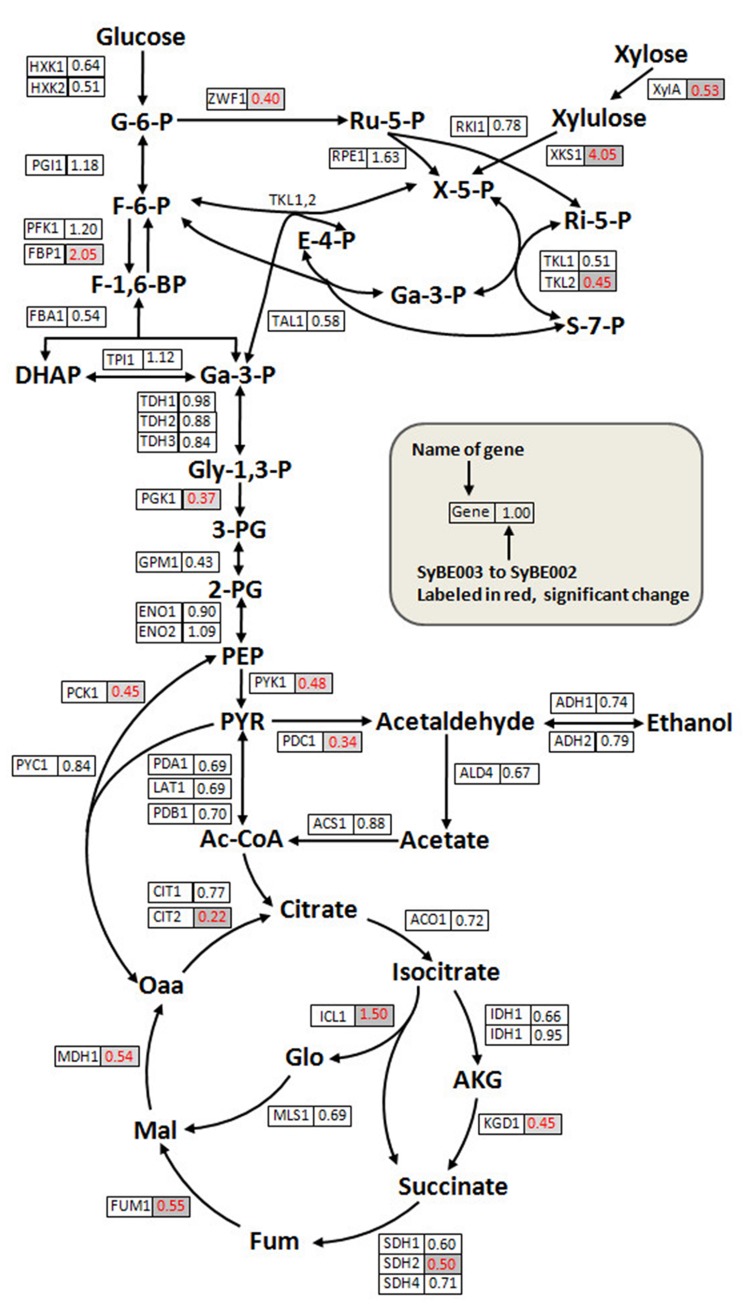
**Quantitative analysis of transcriptional levels of genes in the xylose metabolic pathway and the central carbon metabolism pathway.** Cells of SyBE002 and SyBE003 were cultivated at 20 g/L xylose and harvested after 18 h of fermentation for transcriptional analysis. The name of each gene is shown on the left side of the boxes. The values on the right side of the boxes indicate the ratios of transcriptional levels in SyBE003 to those in SyBE002.

No mutations were observed in the promoters and gene sequences of *XylA* and *XKS1*(sequencing primers in Supplementary Table S5). To investigate the transcriptional changes of SyBE002 after evolution, the expression of genes in PPP, glycolysis, and the tricarboxylic acid (TCA) cycle was monitored using real-time quantitative RT-PCR. As shown in **Figure 5** (raw data in Supplementary Data 2), most of the genes in the initial xylose metabolic pathway showed no significant differences in transcription level except *XylA, XKS1, TKL2*, and *ZWF1*.

The expression level of *XylA* in SyBE003 was 0.53-fold of that in SyBE002. Despite transcriptional decrease of *XylA*, the enzymatic activity of XI in SyBE003 (0.255 ± 0.020 U/mg) was comparable to that in SyBE002 (0.263 ± 0.022 U/mg). The underlying mechanism for this inconsistency will be investigated in the following study.

The expression level of *XKS1* increased by threefold in SyBE003 compared to SyBE002, representing the requirement of abundant expression of *XKS1* for efficient xylose metabolism. In the non-oxidative PPP, the expression levels of most genes did not changed significantly except for *TKL2*, which encodes the minor isoform of transketolase. The expression level of *TKL2* was down-regulated in SyBE003 along with the decreased expression of *TKL1* encoding the major isoform of transketolase. Gene *ZWF1* coding for glucose-6-phosphate dehydrogenase was also down-regulated in SyBE003, which was indicative of a depressed activity of the oxidative PPP part in the unique style of xylose metabolism (**Figure 5**).

The key genes *PGK1* and *PYK1* in glycolysis showed lower levels of mRNA transcripts in SyBE003 compared with those in SyBE002 (**Figure 5**). The expression of gene *PDC1* coding for the major isozyme of pyruvate decarboxylase complex, which is responsible for acetic acid and ethanol production, was also down-regulated. Similarly, the genes in the TCA cycle such as *CIT2, KGD1, SDH2, FUM1*, and *MDH1* exhibited dramatically reduced expression in SyBE003, indicating a decreased respiratory response to xylose, which was also reported in recombinant xylose-fermenting yeasts (Jin et al., 2004). On the contrary, the transcripts for the gluconeogenic enzymes *ICL1* and *FBP1* in SyBE003 were induced to 1.50- and 2.05-fold of the levels in SyBE002, respectively.

Overall, comparatively transcriptional profiling showed that the improved xylose fermentation involved decreased transcription burden of *XylA*, rearrangement of PPP, decreased glycolysis activity, repressed respiration activity, and enhanced gluconeogenesis.

### Effect of *TKL2* Disruption on Xylose Fermentation

**FIGURE 6:**
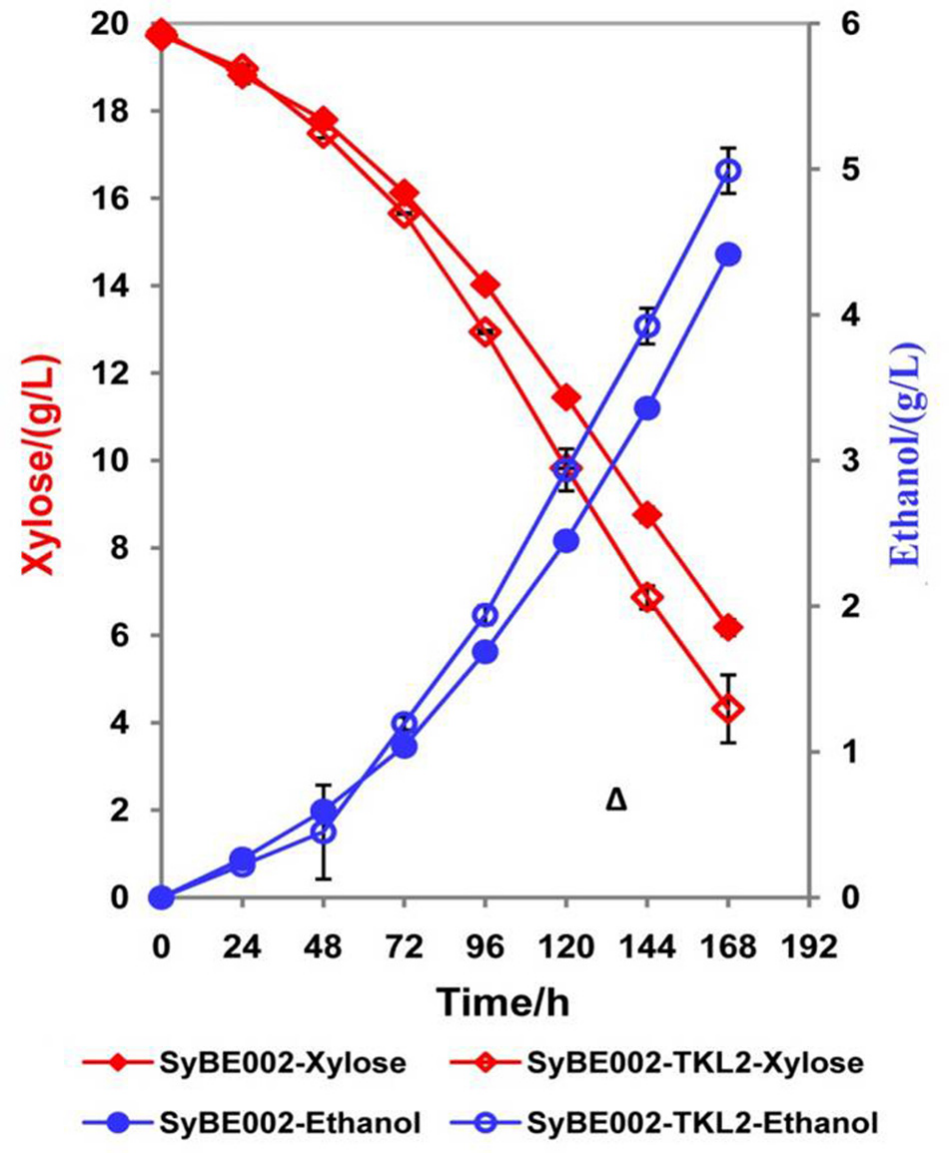
**Deletion of TKL2 accelerates xylose fermentation in SyBE002.** Strain SyBE002-TKL2Δ, in which gene TKL2 was knocked out, and SyBE002 were cultivated in YPX medium with 20 g/L xylose under anaerobic conditions. The initial cell density of inocula was calibrated to 1.0 of OD600. The results shown were the mean value of duplicate experiments. The calculation of xylose consumption rate was performed at 168 h.

To confirm the positive effect of decreased expression of *TKL2*, this gene was knocked out in SyBE002 and the resulting strain SyBE002-TKL2Δ was compared with SyBE002 in terms of xylose fermentation (**Figure 6**). The results showed that SyBE002-TKL2Δ consumed 13.0% more xylose than SyBE002 (**Figure 6**). The final ethanol production of SyBE002-TKL2Δ was 13.0% higher than that of SyBE002. The ethanol, xylitol, and glycerol yields in SyBE002-TKL2Δ were nearly identical to those in SyBE002. The results demonstrated that the decreased expression of *TKL2* did improve xylose fermentation. Single disruption of *TKL2* did not achieve a xylose-fermenting strain as efficient as SyBE003, indicating that the enhanced phenotype was associated with multiple genes besides *TKL2*.

## Discussion

In the present study, we constructed an efficient xylose-fermenting *S. cerevisiae* SyBE003 through the combinatorial expression of key genes and evolutionary engineering. The strain SyBE003 can rapidly utilize xylose and produce ethanol with high yield. Initial rational design of the expression of *XylA* and *XKS1* led to an optimal combination, in which *XylA* and *XKS1* were expressed by *TDH3p* and *TDH1p*, respectively. Combinatorial modulation of expression of multiple genes through promoter shuﬄing is a widely used strategy to optimize metabolic pathways. Lu and Jeffries (2007) used MGPS (multiple-gene-promoter shuﬄing) to successfully optimize the expression of *TAL1, TKL1*, and *PYK1* for improved xylose fermentation. The 15000-fold increased taxadiene production was achieved in a recombinant *E. coli* through combined fine-tuning of the expression of genes in the mevalonic acid pathway and taxadiene biosynthesis pathway (Ajikumar et al., 2010). In this study, combinatorial optimization of *XylA* and *XKS1* improved xylose utilization to some extent. Gene *XKS1* showed a more important role in regulation of xylose utilization during the optimization process. In a previous study, increased expression of *XKS1* improved cell growth and viability when *S. cerevisiae* was cultivated on xylulose (Richard et al., 2000). Overexpression of *XKS1* in the *XYL1* and *XYL2*-expresssing *S. cerevisiae* increased xylose consumption rate by more than onefold under anaerobic conditions (Toivari et al., 2001). The positive effect of *XKS1* overexpression was also observed in a recombinant *S. cerevisiae* expressing *XylA* (Lee et al., 2012). However, excessive overexpression of *XKS1* can also bring adverse effects on xylose metabolism and slow down xylose uptake, which has been reported (Jin et al., 2003; Matsushika and Sawayama, 2008).

Evolutionary engineering is an easy and effective method to reprogram the xylose metabolic pathway amid the limited understanding of transcriptional and post-transcriptional regulation of xylose metabolism so far. Many studies have reported the success of evolutionary engineering in improving xylose utilization. Through evolutionary engineering, improved utilization of xylose and arabinose was obtained after chemostat adaptation of ∼1800 h (Garcia Sanchez et al., 2010). The aerobic growth rate of xylose-fermenting yeast increased by threefold after adaptive evolution (Liu and Hu, 2010). In the present study, the use of evolutionary engineering for xylose utilization improvement is directly related to the growth rate. It was reported that evolutionary engineering of growth-coupled metabolic engineering designs can lead to a significant increase in the production rate and a reduction in byproduct formation (Fong et al., 2005). The evolved strain SyBE003 showed not only significant improvement in growth rate but also an obvious reduction in xylitol production (**Table 2**).

Microorganisms are able to adapt rapidly to different environmental conditions such as carbon source-limited condition. Under a given environment, deleterious and beneficial mutations might occur. The deleterious mutations will be removed by the selective pressure from the environment and accumulation of beneficial mutations will occur. The evolution patterns are hard to predict and random factors such as founder events greatly determine the trait of the population (Simões et al., 2008; Kolbe et al., 2012). Different colonies might have different mutations that can contribute to improved xylose-fermenting property. Physiological fitness can be obtained through different shifts in genomic sequence space (Conrad et al., 2011). Comparative systematic analysis of isolates from intermediate steps of the evolutionary process such as expression profile and proteome profile will help us to understand the mechanism of evolutionary procedure.

The comparison between evolved strains and parent strains at transcriptional levels has provided an opportunity for establishing links between genotype and phenotype, which allows for rational approaches to further modifications (Hong et al., 2011). Transcriptional analysis of a recombinant xylose-fermenting strain TMB3399 and the mutant TMB3400 demonstrated that the improved performance involved xylose transport, initial xylose metabolism and PPP (Wahlbom et al., 2003). New putative bottlenecks in xylose metabolism were identified by proteomic analysis (Karhumaa et al., 2009). In this study, through real-time RT-PCR analysis of SyBE002 and SyBE003, some potential bottlenecks were identified in xylose metabolism, which offered new gene targets for further metabolic engineering of the XI pathway.

The transcriptional level of *XylA* was down-regulated in the evolved strain, which might be ascribed to the decrease in copy number because of the integration into the genomic DNA from a multicopy plasmid, about 60 in copy number (Futcher, 1986). In our study, extraction of the plasmid pTDH3XI carrying *XylA* from SyBE003 failed while it was successful from SyBE002. Moreover, *XylA* fragment could be amplified using genomic DNA from as SyBE003 the PCR template (data not shown). Early research has also observed that *XylA* can be recombined into chromosome and duplicated in evolved strains (Zhou et al., 2012). These results suggested that integration of *XylA* into chromosome might happen during the adaptation. Reducing the transcriptional burden has been an important means to optimize the metabolic pathway (Dueber et al., 2009; Ajikumar et al., 2010; Bond-Watts et al., 2011). Thus, efficient xylose utilization requires optimal expression of *XylA*. Alper and co-workers developed a *XylA* mutant (E15D, E114G, E129D, T142S, A177T, and V433I) through directed evolution, which was 77% more active than the wild type (Lee et al., 2012). Expression of the mutant by a single copy plasmid improved ethanol production by 88%. Expression of the *XylA* mutant could achieve a high enough XI activity under a low transcript level, which could additionally improve xylose fermentation.

Comparison of gene expression also indicated reprogramming of *TKL*. PPP has a lower flux capacity than glycolysis, which contributes to the lower consumption rate of xylose than that of glucose (Kuyper et al., 2005a; Chu and Lee, 2007; Matsushika et al., 2009; Shen et al., 2015). Thus, metabolic engineering of xylose metabolism often involves overexpression of genes in the non-oxidative PPP. In this study, the enhanced expression of *RPE1, RKI1, TKL1*, and *TAL1* indeed increased xylose consumption rate (**Figure 2**). In PPP, *TKL1* is important for the production of the precursors needed for the biosynthesis of aromatic amino acids and nucleic acids. However, excessive expression of *TKL1* can cause the imbalance between PPP and the glycolytic pathway (Matsushika et al., 2012). The expression level of *TKL1*, which was under the control of the strongest promoter *TDH3*, might be too high in this study. Therefore, *TKL1* and *TKL2* were down-regulated to keep the balance of PPP and the glycolytic pathway during the evolution process. Metzger observed that overexpression of the transketolase-encoding gene from *Scheffersomyces stipitis* caused slow xylose consumption in a xylose-utilizing *S. cerevisiae* (Metzger and Hollenberg, 1994). The rates of xylose consumption and ethanol production were slightly impaired when *TKL1* or *TKL2* was over-expressed in the recombinant yeast expressing *XYL1/XYL2/XKS1* (Matsushika et al., 2012). So far, fine-tuning the expression of genes in PPP has never been reported. The result reported in this study indicates that fine-tuning the expression of a gene such as *TKL* may be an important alternative to optimize xylose metabolism in recombinant *S. cerevisiae*.

Besides reprogramming of PPP, the transcriptional levels of *PGK1* and *PYK1* were down-regulated in SyBE003. Pyruvate kinase, encoded by *PYK1*, catalyzes ATP generation in glycolysis and is a sensor of the cytosolic ATP/ADP ratio (Larsson et al., 2000). 3-phosphoglycerate kinase, encoded by the gene *PGK1*, is another enzyme catalyzing the formation of ATP in glycolysis. Expression levels of *PYK1* and *PGK1* are indicators of ATP concentration in cells. ATP is a co-substrate of many kinases such as XK and any imbalance between ATP consumption and generation will decrease metabolic fluxes and eventually shut down the pathway. In SyBE002 the xylose metabolism was restricted and excessive ATP might exist, causing the imbalance of ATP pool. Thus, the decrease in expression of *PYK1* and *PGK1* in SyBE003 might achieve the balance of ATP pool under anaerobic xylose fermentation. Moreover, the increase of *XKS1* expression was observed in SyBE003, whose enzyme product XK consumed ATP. This is consistent with a previous study showing that a higher ATP level was observed in the strain with slower xylose consumption (Toivari et al., 2001).

Although potential bottlenecks were identified in PPP, glycolysis, gluconeogenesis, and the TCA cycle in the present study, other limitations apart from these pathways need clarification. Many transcription factors have been reported to be associated with the regulation of non-favored sugars such as xylose, arabinose, and galactose (Salusjarvi et al., 2008; Turcotte et al., 2010). In one study, the transcription factor *RGT1* responsible for regulation of *HXT* genes showed higher expression in xylose-grown yeast cells than those grown on glucose, providing possible clues in engineering xylose transportation for improved xylose utilization (Salusjarvi et al., 2008). In another instance, the expression of a mutant transcription factor *RAS2* (Tyr112) increased growth rate on galactose in *S. cerevisiae* (Hong et al., 2011). Therefore, global identification of metabolic bottlenecks in xylose metabolism will provide a comprehensive understanding of xylose metabolism and regulation.

The xylose-fermenting *S. cerevisiae* SyBE003 is derived from a laboratory strain L2612 of limited resistance to multiple inhibitors present in lignocellulosic hydrolysates. These inhibitors will greatly restrain cell growth and ethanol productivity especially during the period of xylose consumption (Palmqvist and Hahn-Hagerdal, 2000; Zhu et al., 2015). Increasing the tolerance to multiple inhibitors is the urgent challenge to construct a robust strain that can efficiently produce ethanol from biomass hydrolysates.

## Conclusion

We obtained an efficient xylose-fermenting strain SyBE003 through the combinatorial design of key genes and evolutionary engineering. The optimization process identified an optimal combination of *XylA* and *XKS1.* Evolutionary engineering is a feasible approach to rapidly reconstruct an efficient strain for xylose fermentation, and the xylose utilization rate increased about 10-fold during the adaptation.

## Conflict of Interest Statement

The authors declare that the research was conducted in the absence of any commercial or financial relationships that could be construed as a potential conflict of interest.
