# Supplementary material for: Heterologous xylose isomerase pathway and evolutionary engineering improve xylose utilization in Saccharomyces cerevisiae
Source: Front Microbiol. 2015 Oct 21;6:1165. doi: 10.3389/fmicb.2015.01165 (PMC4612707; doi:10.3389/fmicb.2015.01165)
Supplement: Supplementary file 1 [file Data_Sheet_1.DOCX]

**Table S1 – Information of XylA*.***

| **Gene name** | **Protein source** | **Gene sequence** |
| --- | --- | --- |
| ***XylA*** | ***Piromyces sp. E2***  **(ATCC 76762)** | ***atggccaaagaatacttcccacaaatccaaaagatcaagttcgaaggtaaggattccaagaatccattggctttccattactacgacgccgaaaaagaagttatgggtaagaaaatgaaggactggtagatttgctatggcttggtggcatactttgtgtgctgaaggtgctgatcaatttggtggtggtacaaaatctttcccatggaatgaaggtactgatgctattgaaatcgccaagcaaaaagttgatcggtttcgaaatcatgcaaaaattgggtatcccttactactgcttccacgatgttgatttggtttctgaaggtaactcatcgaagaatacgaatctaacttgaaggctgttgtcgcctacttgaaagaaaagcaaaaagaaaccggtatcaagttgttgtggtctactgctaatgttttcggtcacaagagatatatgaacggtttctaccaacccagattttgatgttgttgctagagccatcgtccaaatcaagaatgctattgatgccggtattgaattgggtgctgaaaattatgttttctggggtggtagagaaggttacatgtctgttgaacaccgaccaaaaaagagaaaaagaacatatggccaccatgttgactatggctagagattatgctagatccaagggttttaagggtactttcttgattgaacctaagccaatggaacctatagcaccaatacgatgttgatactgaaaccgctatcggttttttgaaggctcataacttggacaaggacttcaaggttaacatcgaagtcaaccatgctactttggctggtcatactttcgaacataatggcttgtgctgttgatgctggtatgttgggttctattgatgctaatagaggtgactaccaaaacggttgggatactgatcaattcccaatcgatcaatacgaattggttcaagcctggatggaattatagaggtggtggttttgttaccggtggtactaattttgatgccaagactagaagaaactccaccgatttggaagatatcattattgctcacgtttccggtatggatgctatggcaagagcttggaaatgctgctaagttgttgcaagaatctccatacaccaagatgaagaaagaaagatacgcttctttcgattccggtattggtaaggatttcgaagatggtaaattgaccttggaacaagtctacaatacgtaaaaagaacggtgaacctaagcaaacttccggtaagcaagaattatacgaagctatcgttgccatgtaccaatgataa*** |

**Table S2 - Primers used in the construction of recombinant *S. cerevisiae.***

| **Primers** | **Sequences** |
| --- | --- |
| ***XylA*/*XKS1*** |  |
| *XylA*-TDH3-F | gggAAGCTTAACACGCTTTTTCAGTTCGAGT |
| *XylA*-TDH3-R | gggCTGCAGTTTGTTTGTTTATGTGTGTTTATTCG |
| *XylA*-HXK2-F | gggAAGCTTACGCCATAGAAGAGCAATTTC |
| *XylA*-HXK2-R | gggCTGCAGTTTATTTAATTAGCGTACTTATTATGTGTG |
| *XylA*-PGK1-F | gggAAGCTTGATTCCTGACTTCAACTCAAGACG |
| *XylA*-PGK1-R | gggCTGCAGTGTTTTATATTTGTTGTAAA |
| *XKS1*-PGK1-F | cgcGGATCCGATTCCTGACTTCAACTCAAGACG |
| XKS1-PGK1-R | cggACTAGTTGTTTTATATTTGTTGTAAA |
| XKS1-TDH3-F | cgcGGATCCAACACGCTTTTTCAGTTCGAGT |
| XKS1-TDH3-R | cggACTAGTTTTGTTTGTTTATGTGTGTTTATTCG |
| XKS1-TDH1-F | cgcGGATCCTGCTCATTTACACTCTATATCACCAT |
| XKS1-TDH1-R | cggACTAGTTTGTTTTGTGTGTAAATTTAGTGAAGT |
| XKS1-F | cgcGGATCCATGTTGTGTTCAGTAATTCAGAGAC |
| XKS1-R | CTATCGATTTCAATTCAATTCAATTTAGATGAGAGTCTTTTCCAGTTC |
| XKS1-PGK1t-F | ATTGAATTGAATTGAAATCGATAG |
| XKS1-PGK1t-R | ccgGAATTCAACGAACGCAGAATTTTCG |
| XI-R | GCATGGTTGACTTCGATGTTAAC |
| **RPE1/TAL1** |  |
| TDH1p-F | cgcGAGCTCTGCTCATTTACACTCTATAT |
| TDH1p-R | GCTATAATTGGTTTGACCATTTGTTTTGTGTGTAAATTTAG |
| RPE1-F2 | CTAAATTTACACACAAAACAAATGGTCAAACCAATTATAGC |
| RPE1-R2 | TTTCAATTCAATTCAATTTACTAATCTAGCAAATCTCTAG |
| PGK1t-F3 | CTAGAGATTTGCTAGATTAGTAAATTGAATTGAATTGAAA |
| PGK1t-R3 | TTGAGTTGAAGTCAGGAATCAAGCTTAACGAACGCAGAATTTTCGA |
| PGK1p-F2 | TCGAAAATTCTGCGTTCGTTAAGCTTGATTCCTGACTTCAACTCAA |
| PGK1p-R2 | GTGAGCCGTCGCTAGGACCTTG |
| PGK1p-F3 | AGAAATTACCGTCGCTCGTGAT |
| PGK1p-R3 | CTTTTGAGCTGGTTCAGACATTGTTTTATATTTGTTGTAAAAAG |
| TAL1-F2 | CTTTTTACAACAAATATAAAACAATGTCTGAACCAGCTCAAAAG |
| TAL1-R1 | GATTTCAATTCAATTCAATTTATTAAGCGGTAACTTTCTTTTC |
| PGK1t-F2 | GAAAAGAAAGTTACCGCTTAATAAATTGAATTGAATTGAAATC |
| PGK1t-R2 | ccgCTCGAGAACGAACGCAGAATTTTCGA |
| **RKI1/TKL1** |  |
| PGK1p-RKI-F1 | ccgCTCGAGTATTTTAGATTCCTGACTTC |
| PGK1p-RKI-R1 | TTGGGACACCGGCAGCCATTGTTTTATATTTGTTGTAAAAAG |
| RKI-F2 | CTTTTTACAACAAATATAAAACAATGGCTGCCGGTGTCCCAA |
| RKI-R2 | GATTTCAATTCAATTCAATTTATCACTTTTCGGTAACTTCAAC |
| PGK1t-F3 | GTTGAAGTTACCGAAAAGTGATAAATTGAATTGAATTGAAATC |
| PGK1t-R3 | TGAAAAAGCGTGTTTTTTATGAATTCAACGAACGCAGAATTTTCGA |
| TDH3p-F4 | TCGAAAATTCTGCGTTCGTTGAATTCATAAAAAACACGCTTTTTCA |
| TDH3p-R4 | ATGTCAGTGAATTGAGTCATTTGTTTGTTTATGTGTGTTTATTC |
| TKL1-F5 | GAATAAACACACATAAACAAACAAATGACTCAATTCACTGACAT |
| TKL1-R5 | GATTTCAATTCAATTCAATTTATTAGAAAGCTTTTTTCAAAG |
| PGK1t-TKL1-F6 | CTTTGAAAAAAGCTTTCTAATAAATTGAATTGAATTGAAATC |
| PGK1t-TKL1-R6 | cggGGTACCAACGAACGCAGAATTTTCGAG |
| TKL2-F  TKL2-R | TCAGGCAGCAAGTGACCAT  TGGCTTGTCCCCTCTTACTG |

Underlined sequences are recognition sites of restriction enzyme.

**Table S3 - Primers used in real-time RT-PCR.**

| **Primers** | **Sequences** |
| --- | --- |
| XI-F | GTTTGATTTAGGGTTCATTGGA |
| XI-R | AACGCAGTGGGCTTCTTTT |
| XKS1-F | CAAATCGGTGGAGTTTCTTCT |
| XKS1-R | ATGGATTCTGAGGTTGCTGC |
| RKI-F | AACGCAGTGGGCTTCTTTT |
| RKI-R | AGCCGACCCAGGTGTTATTT |
| RPE1-F | ATTTAGATTCGCCGCTGATGT |
| RPE1-R | AGGTCAAGGTATCTCCAACGC |
| TAL1-F | ACTTCCCAACCGTAGGCTT |
| TAL1-R | GACGATGCTCTGGCAACAAG |
| TKL1-F | GCGGCTAACCTAACGGCTC |
| TKL1-R | CCCAACAACTGAAATGTCTCG |
| TKL2-F | CGATAGCGTCGCCGTGTA |
| TKL2-R | GATGCTGGTATGTTGGGTTCTA |
| ACO1-F | TCCGCTGGTTTACCACAGGTT |
| ACO1-R | CGGAACCTGGCTTCCAGAAA |
| ACS1-F | GCAGGCCCTCCTTCCAGAAC |
| ACS1-R | GCACTTGTGCCACTTGACAAAC |
| ACS2-F | TCCCGACAAGCCAGCTTTGA |
| ACS2-R | CGAGCCACAGCCAACATAGC |
| ADH1-F | TACGCCGGTATCAAATGGTT |
| ADH1-R | TCGTGGGTGTAACCAGACAA |
| ADH2-F | ACTGGCCATTGCCAACTAAG |
| ADH2-R | ACCATTTGATACCGGCGTAG |
| ALD4-F | CGACCCTATTGACAGGGGTAAGG |
| ALD4-R | GCAAAGCCAGCAGAAGATTTCAA |
| PYK1-F | CCCACCAAACCACGAAATGAT |
| PYK1-R | CGTTCAAAGCCTTGACCTTCA |
| CIT1-F | AAGGGAACTACCAAAGGCTGAGG |
| CIT1-R | TGCGCCATTGGATGTAGATCTTT |
| CIT2-F | TGCTAACTGGCGAGGTTCCAA |
| CIT2-R | GCAAACTTTGACTCGCTTTCCA |
| ENO2-F | GCTGACTTGGTTGTCGGTTT |
| ENO2-R | ACAGCCTTGTCACCCAATTC |
|  |  |
| FBA1-F | TTGCCATGGTTCGATGGTATG |
| FBA1-R | TGGTCCATAGCGGCCATTCT |
| FBP1-F | ACACGGGCTCATACGCAGTG |
| FBP1-R | GGCATAGCAAGCGGCTACCA |
| FUM1-F | TGGTTGTTTTCCAAACGGGTTC |
| FUM1-R | TGGCAGCGATATGCATGACAG |
| GND1-F | CCCATTCGCAACATGGTTCA |
| GND1-R | TTCTGGTGTCTCCGGTGGTG |
| GND2-F | CCGGACACTAACAGACGCTACG |
| GND2-R | TGGCTCACCGTTTGATTTGG |
| GPM1-F | GGCGATCATGACGGTCTTACC |
| GPM1-R | CTCCCCCAATCGACGCTTCT |
| GPM3-F | GCCTGTGTTGCAAACCTGGAG |
| GPM3-R | TGTGGAGGACCCCTGATCATT |
| HXK1-F | CCTTTGACACCACTCAATCCAA |
| HXK1-R | TGTTTTGGGAAGCTGGGTACG |
| HXK2-F | GGTGACCGTACCTTTGACACCA |
| HXK2-R | GAAACCCAATGGAATTGGCTCA |
| ICL1-F | TTCTGGTTGGCAGTGTTCATCA |
| ICL1-R | GGGCACCCATCTCATCGAGT |
| IDH1-F | GCCAATGTGGCCCTTTTCAA |
| IDH1-R | GGCAAATCTGGCGATCCTTTC |
| KGD1-F | GGGACCGCAATGACTGGTTC |
| KGD1-R | GAGTCAATTCCGGAGGAACAGG |
| KGD2-F | AGGACGAGCTGTTGGCCACT |
| KGD2-R | CCTCAGCAGGTGCTTCACCA |
| LAT1-F | TGCCGTCTATGTGGAGGACAA |
| LAT1-R | TTGAGGAGCAGCAACGTCAGA |
| MDH1-F | CAAGCATCGTTCGCGATTTG |
| MDH1-R | TGAGATGAATCTGGCGGCTCT |
| MLS1-F | CGCTTTGAATGCTCCTGTGAA |
| MLS1-R | GCAAGTTGGCCACATTTCCA |
| PCK1-F | TCGATGCATTTGCAGGATGG |
| PCK1-R | GGGAACTGACCAGCGTTCCA |
| PDA1-F | GGTCAGGAGGCCATTGCTGT |
| PDA1-R | CTGGAGCGTAAAGGTGCATGG |
| PDB1-F | GTGCCGCTTTGAAGGGTTTG |
| PDB1-R | AACACCCACTGCAGCACCATT |
| PDC1-F | TGTTGTTGCACCACACCTTGG |
| PDC1-R | TGGGACGTTCAAGTCGACCA |
| PFK1-F | ACCAACGAAACCGCCACAAT |
| PFK1-R | GCGGAATCGGTTGCATTAGC |
| PGI1-F | CCACGTCGCATTGAGAAACAG |
| PGI1-R | GACCCAAATCGGAACCACCA |
| PGK1-F | TTCTGCCCCAGGTTCCGTTA |
| PGK1-R | TGAGCGGTACCGAAGGCATC |
| PYC1-F | TGAAGGCCGGTATCACTTGGA |
| PYC1-R | GATCACCGGGTAGCCGTATTCA |
| SDH1-F | AGGTGACCAGGACTCCATCCA |
| SDH1-R | AGGCCTGAGCACCCTTACCG |
| SDH2-F | CGACCAGAACGAATCCAAACAA |
| SDH2-R | GCACTTCCGTTCCATCCTTTG |
| SDH4-F | TGGTGTTTGGCACAAGTACGC |
| SDH4-R | TGGCTTCAATCTTTTGACTGTTG |
| TDH1-F | CTCACGCTTCCATCTTCGAT |
| TDH1-R | AAGCCTTGGCAACATATTCG |
| TDH2-F | CGTCGAAGTTGTTGCTTTGA |
| TDH2-R | TGTCATCGTGGGAAACTTCA |
| TDH3-F | GTTGCTTTGAACGACCCATT |
| TDH3-R | GCTTGTCATCGTGGGAAACT |
| TPI1-F | GTTCGCTTTAGGTCAAGGTGTCG |
| TPI1-R | AGCAGCCAAACCGGTACCAA |
| ZWF1-F | AAAGTGCCAACGTCGATGTCC |
| ZWF1-R | AAGAGGGGCCCCAGGTTTTT |
| ENO1-F | AAGGTGGTGTTGCTCCAAAC |
| ENO1-R | GCACAGTCCAAACCGATCTT |
| ACT1-F | AGCCGACCCAGGTGTTATTT |
| ACT1-R | ATTTAGATTCGCCGCTGATGT |

**Table S4 Performance of strains harboring different modules of XylA-XKS1.**

|  | average | | | | standard deviation | | | |
| --- | --- | --- | --- | --- | --- | --- | --- | --- |
| Xylose comsumption (g/L) |  | TDH1 | PGK1 | TDH3 |  | TDH1 | PGK1 | TDH3 |
|  | PGK1 | 11.14 | 6.87 | 7.49 | PGK1 | 0.29 | 0.75 | 0.09 |
|  | TDH3 | 12.32 | 7.70 | 8.38 | TDH3 | 0.00 | 0.31 | 0.13 |
|  | HXK2 | 11.32 | 7.84 | 7.73 | HXK2 | 0.24 | 0.08 | 0.19 |
|  |  |  |  |  |  |  |  |  |
| Xylitol production (g/L) |  | TDH1 | PGK1 | TDH3 |  | TDH1 | PGK1 | TDH3 |
|  | PGK1 | 0.74 | 1.30 | 1.25 | PGK1 | 0.01 | 0.04 | 0.00 |
|  | TDH3 | 0.70 | 1.20 | 1.15 | TDH3 | 0.01 | 0.02 | 0.01 |
|  | HXK2 | 0.75 | 1.22 | 1.23 | HXK2 | 0.01 | 0.01 | 0.00 |
|  |  |  |  |  |  |  |  |  |
| Dry Cell Weight (g/L) |  | TDH1 | PGK1 | TDH3 |  | TDH1 | PGK1 | TDH3 |
|  | PGK1 | 3.54 | 3.30 | 3.31 | PGK1 | 0.02 | 0.01 | 0.09 |
|  | TDH3 | 4.50 | 3.15 | 3.21 | TDH3 | 0.26 | 0.06 | 0.01 |
|  | HXK2 | 3.96 | 3.31 | 3.35 | HXK2 | 0.28 | 0.08 | 0.03 |

**Table S5 - Primers used in the gene sequencing after adaptation.**

| **Primers** | **Sequences** |
| --- | --- |
| THD3-PGK1-F  THD3-PGK1-R  THD1-PGK1-F  THD1-PGK1-R | CAGTTCGAGTTTATCATTATCAA  CGAAAATTCTGCGTTCGTT  GTGATATAGAGTGTAAATGAGCATATACA  CGAAAATTCTGCGTTCGTT |
